# Role of Endothelial Nitric Oxide Synthase in Diabetic Nephropathy: Lessons from Diabetic eNOS Knockout Mice

**DOI:** 10.1155/2014/590541

**Published:** 2014-10-13

**Authors:** Takamune Takahashi, Raymond C. Harris

**Affiliations:** Division of Nephrology and Hypertension, Vanderbilt University School of Medicine, S-3223, Medical Center North, Nashville, TN 37232, USA

## Abstract

Diabetic nephropathy (DN) is the leading cause of end-stage renal disease in many countries. The animal models that recapitulate human DN undoubtedly facilitate our understanding of this disease and promote the development of new diagnostic markers and therapeutic interventions. Based on the clinical evidence showing the association of eNOS dysfunction with advanced DN, we and others have created diabetic mice that lack eNOS expression and shown that eNOS-deficient diabetic mice exhibit advanced nephropathic changes with distinct features of progressive DN, including pronounced albuminuria, nodular glomerulosclerosis, mesangiolysis, and arteriolar hyalinosis. These studies clearly defined a critical role of eNOS in DN and developed a robust animal model of this disease, which enables us to study the pathogenic mechanisms of progressive DN. Further, recent studies with this animal model have explored the novel mechanisms by which eNOS deficiency causes advanced DN and provided many new insights into the pathogenesis of DN. Therefore, here we summarize the findings obtained with this animal model and discuss the roles of eNOS in DN, unresolved issues, and future investigations of this animal model study.

## 1. Introduction

Diabetic nephropathy (DN) is the single major cause of renal failure in many countries. In the past decades, a variety of rodent models have been created and utilized to investigate this disease [1]. These studies have given many new insights into the pathogenic pathways of DN and facilitated the development of therapeutic agents for this disease. However, most of the models displayed early nephropathic changes, including mild albuminuria, hyperfiltration and hypertrophy, mesangial expansion, and thickening of glomerular basement membrane (GBM), and the advanced nephropathic changes such as pronounced albuminuria, decline of GFR, and glomerular mesangiolysis and nodular glomerulosclerosis were rarely observed in these models. Thus, the lack of robust animal models of DN made it difficult to investigate the pathological mechanisms underlying advanced DN and to evaluate the effects of pharmacologic agents for progressive DN.

Although multiple cell types are involved, DN is in essence a microvascular disease that develops as a result of a confluence of hemodynamic and metabolic perturbations. There is compelling evidence that endothelial dysfunction serves as a key event in the development and progression of diabetic vascular complications, including nephropathy [2–4]. Endothelial cells maintain vascular function and homeostasis by generating paracrine factors that regulate vascular tone, preventing coagulation and platelet aggregation, inhibiting adhesion of leukocytes, and limiting proliferation of vascular smooth muscle cells as well as by constituting a selective barrier to the diffusion of macromolecules into the interstitial space. Further, it was shown that nitric oxide (NO) produced by endothelial cells through the endothelial nitric oxide synthase (eNOS) plays a major role for many of these endothelial functions [5] and that decreased NO production and bioavailability largely contribute to endothelial dysfunction in diabetes [2, 3].

In the past decades, a large body of experimental studies has suggested that development and/or progression of DN is associated with alterations in eNOS expression and activity [2]. eNOS was shown to be the major NOS enzyme in renal vasculature [6–8], and eNOS expression was shown to be upregulated in early (1–6 weeks) diabetic kidneys, especially in afferent and glomerular endothelium, concomitant with increases in diameter of afferent arterioles, glomerular volume and filtration rate, and urinary NO metabolites (NO_x_) [9–11]. Further, NADPH diaphorase staining suggested that eNOS activity is increased in afferent artery and glomerular endothelium in diabetic kidney [10]. On the other hand, the studies assessing NO production or responses in renal vasculature and glomerulus demonstrated decreased eNOS and NO activity in DN, even when eNOS expression is upregulated [12–15]. The eNOS uncoupling caused by reactive oxygen species has been suggested to be a mechanism underlying this paradox [15]. Further, a triphasic response of increased, unaltered, and impaired endothelial NO-dependent vascular relaxation within the same diabetic animal [16] and the downregulation of glomerular or renal eNOS expression in progressive models of DN (OVE26 mouse, ZSF_1_ rat) [17, 18] suggested that eNOS-mediated NO production is decreased during the progression of DN. In this context, it is noteworthy that a nonselective NOS inhibitor attenuates the early renal vasodilatation and hyperfiltration in diabetic animals [19, 20], while its chronic treatment remarkably aggravates diabetic glomerular injury [21]. In aggregate, these findings suggest that eNOS serves as a key mediator in the development and progression of DN.

In humans, upregulated eNOS expression in glomerular endothelium was demonstrated in nephropathy patients with type 2 diabetes [22, 23]. Interestingly, macroalbuminuric patients showed lower glomerular eNOS expression than microalbuminuric patients [22]. Further, urine or serum NO metabolites (NO_2_^−^ + NO_3_^−^) were shown to be increased in normo- or microalbuminuric diabetes patients, associated with increases in glomerular filtration rate (GFR) [22, 24–26], while preferential changes of plasma NO_3_^−^ in macroalbuminuric patients suggested decreased NO bioavailability in these patients [27]. It is of interest that African and Asian type II diabetic patients, who are susceptible to end-stage renal failure, exhibited lower NO production compared to a comparable group of Caucasian diabetic patients [28]. Last, genetic association studies have shown that eNOS polymorphisms that potentially impair eNOS gene transcription and activity are associated with an increased risk of advanced DN [29, 30]. Collectively, these experimental and clinical evidences suggest that renal eNOS expression and activity are increased early after the onset of diabetes, possibly mediating vasodilatation and hyperfiltration; however, they are decreased with prolonged diabetes and the resulting vascular NO deficiency may facilitate the progression of DN. Multiple factors and mechanisms have been suggested for the eNOS upregulation or activation (e.g., high glucose, VEGF, IGF-1, and shear stress), for the eNOS dysfunction, inactivation, and downregulation (e.g., ROS, angiotensin II, AMDA, PKC, AGE, and TNF*α*), and for reduction of NO bioavailability (e.g., excessive superoxide production) in DN [2, 31]. Thus, there is substantial evidence that eNOS may serve as a key player in DN. However, the precise role of eNOS in this disease remained unknown.

In the past decade, multiple groups have created the eNOS-deficient diabetic mice to investigate its role in DN and shown that these diabetic animals exhibit advanced nephropathic changes with the features similar to human DN. These studies clearly defined a pivotal role of eNOS in DN and developed a robust animal model of this disease. Further, recent studies with this animal model have explored the novel mechanisms by which eNOS deficiency causes advanced DN and provided many new insights into the pathogenesis of DN. Since this animal model is now widely used for the study of DN, herein we summarize the findings obtained with this animal model and discuss unresolved issues and future investigations.

## 2. Diabetic eNOS Knockout Study

### 2.1. Reported Models

*(a) Nonobese Models*. We [32] and Nakagawa et al. [33] induced diabetes in eNOS −/− male mice (C57BL/6J strain) using either low- (50 mg/kg, 5 days) or high- (100 mg/kg, 2 days) dose streptozotocin (STZ) injections. Li et al. [34] also generated diabetic eNOS −/− mice (C57BL/6J strain) using low-dose STZ injections and fed the mice either with a normal or high-fat diet. In this study, STZ diabetes was induced at 4–6 months of age, while the former studies induced diabetes at 6–8 weeks of age. Further, Wang et al. [35] created a spontaneous model of diabetic eNOS −/− and eNOS +/− mice (C57BL/6J:129S6/SvEvTac F1 hybrid) by introducing the dominant Akita diabetogenic mutation (Ins2^C96Y/+^) to eNOS +/− and eNOS −/− mice.

*(b) Type 2 Diabetes Model*. We [36] and Mohan et al. [37] generated the eNOS-deficient db/db (lepr^db/db^) mice by backcrossing eNOS −/− mice (C57BL/6J strain) onto db/db strain (C57BLKS/J background).

### 2.2. General Characteristics (Table 1)

*(a) Hyperglycemia*. In nonobese models, the blood glucose levels in diabetic eNOS −/− or eNOS +/− mice were comparable to those in diabetic eNOS +/+ mice. In db/db model, db/db eNOS −/− mice developed obesity and diabetes similar to db/db eNOS +/+ mice. There was no difference in the timing of development of diabetes between these two groups [36]. Interestingly, db/db eNOS −/− mice showed significantly increased body weight, lower blood glucose levels, and prominent (~5 fold increase) hyperinsulinemia as compared with db/db eNOS +/+ mice [36, 37]. In addition, prominent islet enlargement and macrovesicular fat droplets were noted in the pancreas or liver in db/db eNOS −/− mice [37]. In all studies, nondiabetic eNOS −/− mice showed normal blood glucose levels.

*(b) Survival Rate*. In high-dose STZ and Akita models, diabetic eNOS −/− or eNOS +/− mice showed relatively lower survival rate (50% at 5-6 months after diabetes) compared with diabetic eNOS +/+ mice (100% survival) [33, 35]. The low-dose STZ eNOS −/− mice in which diabetes was induced at 4–6 months of age also showed lower survival rate (70% at 6 months after diabetes) [34]. In this study, it was also shown that the survival of STZ eNOS −/−, but not STZ eNOS +/+, mice is significantly decreased by high-fat diet. In contrast, in our low-dose STZ study, all STZ eNOS −/− mice survived until 5 months after STZ injections. Thus, it seems that the survival rate of diabetic eNOS −/− mice differs among the models, perhaps due to the dose of STZ or the age of onset of diabetes. Nondiabetic eNOS −/− mice survived well during the study period [33, 35]. For db/db eNOS −/− model, Mohan et al. described that the average lifespan of db/db eNOS −/− mice is 9 months to 1 year without insulin treatment [37], while our db/db eNOS −/− mice only survive for 26–30 weeks.

*(c) Body Weight*. High-dose STZ eNOS −/− mice exhibited severe body weight loss compared with STZ eNOS +/+ mice [33], while this phenotype was not observed in low-dose STZ eNOS −/− and Akita eNOS −/− (3-month old) models. Interestingly, 7-month old Akita eNOS −/− or eNOS +/− mice showed significantly higher body weight than Akita eNOS +/+ mice [35]. In db/db model, db/db eNOS −/− mice showed significantly higher body weight than db/db eNOS +/+ mice [37]. Difference was not observed in body weight between nondiabetic eNOS +/+ and eNOS −/− mice in all the models.

*(d) Hypertension*. In all studies, nondiabetic eNOS −/− or eNOS +/− mice were hypertensive as described previously [38]. Diabetes further increased blood pressure (BP) of eNOS −/− mice in STZ (high- and low-dose) and Akita models [32, 33, 35], while this effect was not observed in db/db model [36, 37] and some low-dose STZ studies [34, 39]. Of importance, in all studies, diabetic eNOS −/− mice showed significantly higher BP (10–48 mmHg) levels than diabetic eNOS +/+ mice (Table 1).

*(e) Hyperlipidemia*. In low-dose STZ study, diabetes or eNOS genotype did not affect plasma triglyceride and total cholesterol levels [34]. It is of note that high-fat diet significantly increased plasma cholesterol levels in low-dose STZ eNOS −/− mice, while its increase was limited in STZ eNOS +/+ mice [34]. Plasma lipid levels are not described in high-dose STZ and Akita eNOS −/− studies. In db/db model, db/db eNOS −/− mice showed significantly higher plasma cholesterol levels compared with db/db eNOS +/+ and nondiabetic controls, while no significant difference was observed in plasma triglyceride levels between db/db eNOS −/− and other groups [37].

### 2.3. Renal Phenotype

#### 2.3.1. Common Renal Phenotype

*(a) Pronounced Albuminuria*. In all models, diabetic eNOS −/− mice exhibited pronounced albuminuria compared with diabetic eNOS +/+ mice and the albuminuria further increased as the disease progressed. The severity of albuminuria appears to differ among the models and prominent albuminuria was observed in high-dose STZ and db/db eNOS −/− mice (Table 2). Nondiabetic eNOS −/− mice also showed significant albuminuria in some [33, 35, 36], but not all, studies, yet its levels were much lower than diabetic eNOS −/− mice, indicating that diabetes is required to cause pronounced albuminuria in eNOS −/− mice. It is of note that overt albuminuria was observed in low-dose STZ eNOS −/− mice as early as at 14 days after STZ injections [39], suggesting that hyperglycemia rapidly induces albuminuria in eNOS −/− mice. A focal podocytopathy was suggested as a mechanism of this. We also observed an increase in albuminuria in low-dose STZ eNOS −/− mice as early as at 6 weeks after STZ injections [32]; however, in other STZ studies, the increases in albuminuria were not observed in STZ eNOS −/− mice at 1.5–3 months after diabetes induction [33, 34]. The reason for this difference is currently unknown. It should also be noted that Akita eNOS +/− mice developed albuminuria at levels comparable to those in Akita eNOS −/− mice [35]. Given the fact that heterozygous deletion of eNOS gene reduces renal eNOS protein levels by ~65% [35], this finding indicates that not only “deficiency” but also “reduction” of eNOS are sufficient to accelerate albuminuria in diabetic mice.

*(b) Advanced Glomerular Lesions*. (1) Light microscopy: compared with diabetic eNOS +/+ mice, diabetic eNOS −/− mice showed advanced glomerular lesions that are similar to what is seen with human DN. These include glomerular mesangiolysis and microaneurysms, advanced mesangial expansion occasionally forming nodular or Kimmelstiel-Wilson like lesion, nodular and global glomerulosclerosis, arteriolar hyalinosis, subendothelial hyaline deposition resembling fibrin caps, glomerular and arteriolar fibrin deposition, and tubulointerstitial fibrosis. Mesangiolysis was occasionally accompanied by arteriolar lesions and intraluminal fibrin deposition [33, 37]. Adhesions to Bowman's capsule were also noted in db/db eNOS −/− mice [37]. It is noteworthy that Akita eNOS +/− as well as eNOS −/− mice exhibited advanced glomerular lesions as compared with Akita eNOS +/+ and nondiabetic eNOS −/− mice, although the lesions in Akita eNOS +/− were milder than Akita eNOS −/− mice [35]. This finding indicates that reduction of eNOS is sufficient to exacerbate histopathological changes of DN. Mesangiolysis, glomerular fibrin deposition, and globally sclerotic glomeruli were also observed in nondiabetic eNOS −/− mice; however, its frequency was much lower than in diabetic eNOS −/− mice [32, 33, 37]. Importantly, the percentage of totally sclerotic glomeruli was 2.8-fold increased in db/db eNOS −/− mice from 16 to 40 weeks of age [37], indicating progressive glomerular injury in this model. It is of note that renal histopathology of diabetic eNOS −/− mice in low-dose STZ and Akita models appears to be milder than those in high-dose STZ and db/db models [32, 39]. Also, glomeruloslerosis and tubulointerstitial fibrosis seem to be more severe in the STZ eNOS −/− mice in which low-dose STZ was injected at older ages [34]. These findings suggest that the dose of STZ, type of diabetes, or age may significantly affect renal injury in diabetic eNOS −/− mice. (2) Electron microscopy: consistent with light microscopic findings, electron microscopy of diabetic eNOS −/− or eNOS +/− glomeruli showed advanced glomerular lesions, including mesangiolysis that is characterized by accumulation of electron-lucent material in the mesangium and dissociation of the mesangial matrix, microaneurysm formation due to disruption of anchoring of the GBM to the mesangium, advanced mesangial expansion occasionally forming lobular or nodular mesangial architecture, and GBM thickening [32–37]. The mesangiolysis accompanied destructive endothelial morphology similar to thrombotic microangiopathy. These include detachment of endothelial cells from the GBM, subendothelial accumulation of electron-lucent material, and intracapillary fibrin deposition and platelet accumulation [32, 37], suggesting advanced glomerular endothelial injury in diabetic eNOS −/− mice. It is of interest that eNOS −/− or eNOS +/− genotype caused striking GBM thickening in Akita model [35] as compared with other models. (3) Immunohistochemistry: diabetic eNOS −/− glomeruli showed increased macrophage infiltrate (CD68, F4/80, or MOMA-2 immunostain) [34, 35, 37, 42, 43], focal loss of endothelial staining (CD31 or CD34 immunostain) [33, 37], fibrin deposition [33–35, 37], and prominent fibronectin [36] and collagen accumulation, whereas these changes were highly limited or not observed in diabetic eNOS +/+ mice and nondiabetic controls. Macrophage influx and endothelial loss were observed mainly in the glomeruli with mesangiolysis [37]. In addition, TUNEL, PCNA, or Ki67 staining demonstrated endothelial apoptosis and proliferation in diabetic eNOS −/− glomeruli and peritubular capillaries [33, 37]. Electron dense deposits or immunoglobulin staining were not observed in diabetic eNOS −/− glomeruli, indicating that glomerular injury is not associated with immune-complex deposition.

#### 2.3.2. Distinct Renal Phenotype (Table 2)

*(a) Glomerular Filtration Rate (GFR)*. In a high-dose STZ model, GFR was remarkably (~65%) decreased in diabetic eNOS −/− mice compared with nondiabetic control [44], while in low-dose STZ models diabetes increased GFR in eNOS −/− mice, although it did not reach the levels in diabetic eNOS +/+ mice [32, 39]. Interestingly, Akita eNOS −/− and eNOS +/− mice showed more pronounced diabetic hyperfiltration than Akita eNOS +/+ mice [35], and the GFR declined in Akita eNOS −/− mice as the disease progressed, showing significantly lower GFR than Akita eNOS +/+ mice at 7 months of age, yet it was still higher than nondiabetic controls. GFR was progressively increased in Akita eNOS +/− mice and its decline was not observed at 7 months of age. Thus, eNOS deficiency showed the opposite effects for diabetic hyperfiltration between STZ and Akita models. It should be noted that Akita eNOS −/− study clearly demonstrated that prolonged eNOS deficiency leads to the decline in glomerular filtration in diabetic mice. db/db eNOS −/− mice showed significantly decreased GFR as compared with db/db or nondiabetic controls at 26 weeks of age [36]. Thus, db/db eNOS −/− mice showed lower GFR than those of nondiabetic control mice, suggesting more severe glomerular injury than in low-dose STZ or Akita eNOS −/− mice.

*(b) Tubulointerstitial Lesions*. STZ eNOS −/− mice exhibited significant tubulointerstitial lesions compared with STZ eNOS +/+ or nondiabetic eNOS −/− mice [34, 44, 45]. Tubular TGF*β* expression and interstitial macrophage infiltrate (F4/80 staining) and collagen deposition were also demonstrated in STZ eNOS −/− mice [40, 41, 45]. However, surprisingly, Akita eNOS −/− or eNOS +/− mice showed significantly decreased tubulointerstitial fibrosis compared with Akita eNOS +/+ mice [35]. db/db eNOS −/− mice showed advanced tubulointerstitial injury as evidenced by destructive tubular morphology and interstitial collagen accumulation [37]. Further, Kim-1, TUNEL, PCNA, and CD68 staining showed increased tubular injury, apoptosis, or proliferation, and interstitial macrophage infiltration in db/db eNOS −/− kidney [37, 41]. db/db eNOS +/+ mice showed highly limited tubulointerstitial lesions. Thus, significant tubulointerstitial lesions were observed in STZ and db/db eNOS −/− mice, while Akita eNOS −/− mice had opposite results.

*(c) Renal Oxidative Stress and Sensitivity to RAS Blockade*. eNOS deficiency did not alter renal oxidative stress in low-dose STZ diabetic mice [34], whereas it was markedly increased in db/db eNOS −/− mice [41]. Surprisingly, Akita eNOS −/− or eNOS +/− kidneys showed less renal oxidative stress than Akita eNOS +/+ or nondiabetic controls [35]. Nephropathy in low-dose STZ or db/db eNOS −/− mice was remarkably suppressed by angiotensin-converting enzyme inhibitor (ACEI) or angiotensin receptor blocker (ARB) [39, 41]. However, these agents were ineffective for high-dose STZ eNOS −/− mice [46].

In summary, as compared with diabetic eNOS +/+ mice, diabetic eNOS −/− or eNOS +/− mice showed advanced nephropathic changes that overlap to human DN, although some differences were observed between the models. Given the fact that a much less severe renal phenotype was observed in nondiabetic eNOS −/− mice, these findings define a critical role of eNOS in the development and progression of DN. The renal phenotype in db/db eNOS −/− mice was more severe than in low-dose STZ or Akita eNOS −/− models. Although the precise mechanism of this is currently unknown, this may be because (1) obesity-related factors, including insulin resistance, hyperinsulinemia, and hyperlipidemia, accelerate the renal injury in diabetic eNOS −/− mice. Indeed, a high-fat diet significantly advanced the renal phenotype in low-dose STZ eNOS −/− mice [34]. Also, hyperinsulinemia was shown to promote vascular inflammation and PAI-1 expression [47–49]. In addition, eNOS deficiency and hyperinsulinemia may induce abnormal endothelial insulin signaling and cause vascular disorders. The insulin receptor transduces two distinct signals in endothelium [50], (i) promoting NO production through the PI3 K/Akt/eNOS pathway and (ii) stimulating endothelial release of vasoconstrictor ET-1 and expression of VCAM-1/E-selection through the MAP kinase cascade. The net effect is negligible in normal subjects. However, in the setting of eNOS deficiency, hyperinsulinemia may largely stimulate the latter endothelial insulin signaling. This imbalanced insulin signaling may promote vasoconstriction (ET-1 release) and vascular inflammation, advancing renal pathology in db/db eNOS −/− mice. (2) The genetic background of db/db eNOS −/− mice causes more advanced DN. The db/db eNOS −/− mice were created with the C57BLKS/J strain, while STZ or Akita eNOS −/− mice studies were carried out with the C57BL/6J strain or C57BL/6J × 129S6/SvEvTac F1 cross. The C57BL/6J strain is known to be relatively resistant to DN comparing with C57BLKS/J strain [51–53]. The C57BL/6J × 129S6/SvEvTac F1 cross also seems to be resistant to DN as albuminuria in Akita eNOS +/+ mice is relatively mild (<100 *μ*g/day). Hence, the more severe renal phenotype in db/db eNOS −/− mice may be associated with its genetic background. Indeed, we have recently noted that C57BLKS/J-strain STZ eNOS −/− mice develop more advanced nephropathy than C57BL/6J-strain STZ eNOS −/− mice [53]. However, the genetic background may not sufficiently explain the severity of DN in db/db eNOS −/− mice as the nephropathy in C57BLKS/J-strain STZ eNOS −/− mice was milder than that in db/db eNOS −/− mice. (3) Hyperleptinemia induced by the db/db mutation in ObRb leptin receptor may modify the renal phenotype in db/db eNOS −/− mice as leptin has been shown to exhibit deleterious renal effects through other leptin receptor isoforms (e.g., ObRa receptor) that is present in glomerular cells, promoting TGF*β* and TGF*β* type II receptor expression and increasing collagen synthesis in glomerular cells [54].

**Figure 1:**
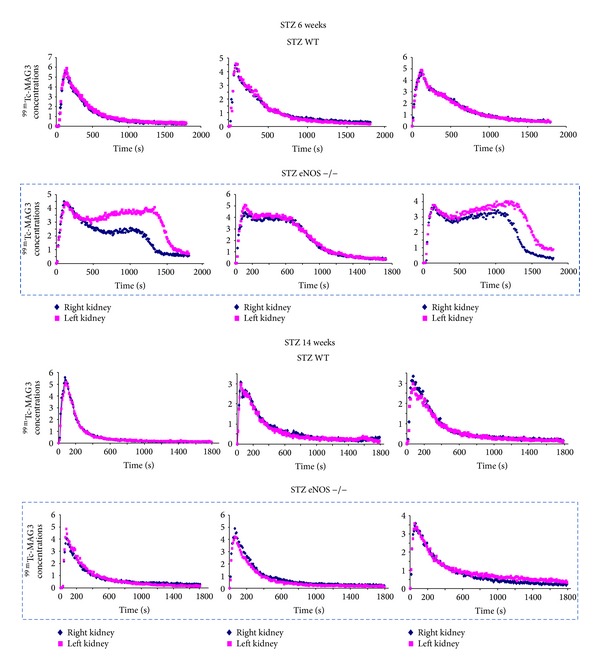
STZ injection causes acute tubular injury in eNOS −/−, but not eNOS +/+, mice. Tubular function of STZ eNOS −/− and eNOS +/+ mice were assessed by ^99m^Tc-MAG3 dynamic renal scintigraphy at 6 and 14 weeks after low-dose STZ injections (50 mg/kg, 5 days). STZ eNOS −/− mice (dashed box) showed delayed ^99m^Tc-MAG3 excretion at 6 weeks after STZ injections, indicating tubular injury in these mice; however, these mice exhibited nearly normal pattern of ^99m^Tc-MAG3 renal scintigraphy at 14 weeks after STZ injections. STZ eNOS +/+ mice showed normal ^99m^Tc-MAG3 renogram at both time points.

It should also be noted that high-dose STZ eNOS −/− mice showed a much more severe phenotype than low-dose STZ or Akita models (Table 2), although there was no difference in the severity of hyperglycemia among these models. This finding suggests that STZ may be highly toxic to eNOS −/− mice. STZ is known to also be taken up by extraislet cells via GLUT2 and cause tissue injury, including liver (hepatocytes) and kidney (proximal tubular cells and possibly podocytes) [55–58]. In kidney, it causes tubular injury and also advances albuminuria, especially when it is used at a high dose [1, 58, 59]. As shown in Figure 1, we have assessed tubular function in low-dose STZ eNOS −/− mice using ^99m^Tc-MAG3 renal scintigraphy [60]. Interestingly, low-dose STZ eNOS −/−, but not STZ eNOS +/+ or nondiabetic control (not shown), mice showed obvious tubular injury at 6 weeks after STZ injections, as evidenced by delayed ^99m^Tc-MAG3 excretion. However, surprisingly, the tubular injury was largely restored in STZ eNOS −/− mice at 14 weeks after STZ injections and the mice showed nearly normal ^99m^Tc-MAG3 renograms up to 35 weeks after STZ injections, suggesting mild or focal tubular injury in this model. This finding suggests that eNOS −/− mice may be more susceptible to STZ tubular toxicity than eNOS +/+ mice, resulting in acute tubular injury, and that this tubular damage is mostly recovered by 14 weeks after STZ injections (when STZ is used at a low dose). The renal phenotype in high-dose STZ eNOS −/− mice was shown to be improved by insulin treatment [33]; however, it may be difficult to exclude STZ renal toxicity with these data as insulin transduces a variety of biological signals in many cell types in addition to lowering blood glucose levels [61]. Also, it is possible that hyperglycemia largely enhances renal toxicity of STZ in eNOS −/− mice; accordingly, normalizing blood glucose levels reduces the STZ renal toxicity as well as diabetic renal injury. Together with the fact that tubular injury is severe in diabetic eNOS −/− mice in the order high-dose STZ > low-dose STZ > Akita model, tubular pathology in STZ eNOS −/− models may in part result from STZ toxicity, even with low dose.

The following points should also be noted with the renal phenotype of diabetic eNOS −/− mice: (1) overt DN was developed by eNOS deficiency in C57BL/6 strain mice that is known to be resistant to DN [51, 52]. This finding indicates that eNOS deficiency is sufficient to develop as well as to progress DN. (2) Despite evident glomerular capillary injury, the aorta was normal in db/db eNOS −/− [37] and low-dose STZ eNOS −/− (Kanetsuna Y, unpublished data) mice. Early or established atherogenic lesions were not observed in these mice. These findings suggest that eNOS mediates differential effects within the vascular system in diabetes. (3) The increases in kidney/body weight ratio or kidney weight in diabetic eNOS −/− mice were comparable to those in diabetic eNOS +/+ mice, except for a high-dose STZ model. This finding suggests a minimal role of eNOS in diabetic renal hypertrophy. The pronounced renal hypertrophy in high-dose STZ eNOS −/− mice may be due to prominent body weight loss of these mice [33].

## 3. Proposed Mechanisms of Advanced DN in Diabetic eNOS −/− Mice

The eNOS-derived NO is known to mediate various biological functions and responses in vasculature and play a major role in vascular homeostasis. These include vasodilatation (antihypertensive), inhibition of platelet aggregation and coagulation (anti-thrombotic), and suppression of inflammation (anti-inflammatory). Further, NO has been shown to prevent endothelial apoptosis induced by high glucose through inhibition of NF-*κ*B pathway (antiapoptotic) [62, 63], suppress cytokine-induced endothelial activation [64] as well as production of cytokines in endothelial cells [65], and inhibit TGF*β*, collagen, and fibronectin production in mesangial or endothelial cells (antifibrotic) [66, 67]. Thus, multiple mechanisms could be involved in the acceleration of glomerular injury in diabetic eNOS −/− mice and the renal phenotype of diabetic eNOS −/− mice seems to be well explained by the disruption of these reported vasoprotective actions of NO. The following pathways or mechanisms have been investigated in more detail.

*Hypertension and Renin-Angiotensin System*. NO negatively regulates blood pressure (BP) and counteracts angiotensin II (Ang II), which are key pathogenic mediators in DN. Therefore, advanced nephropathy in diabetic eNOS −/− mice may be caused by hypertension and/or enhanced action of Ang II. Kosugi et al. have shown that lowering BP with hydralazine largely prevents glomerular injury in high-dose STZ eNOS −/− mice, yet hydralazine treatment was ineffective for attenuating tubulointerstitial lesions [44]. Further, they also showed that ACEI (enalapril) or ARB (telmisartan) is less effective in reducing BP and glomerular injury in STZ eNOS −/− mice, whereas they are effective for STZ eNOS +/+ mice [46]. Interestingly, enalapril or telmisartan increased serum aldosterone levels in STZ eNOS −/− mice, while these agents decreased serum aldosterone in STZ eNOS +/+ mice. Furthermore, the authors demonstrated that aldosterone receptor antagonist (spironolactone) effectively reduces BP and glomerular and tubulointerstitial injury in STZ eNOS −/− mice. These findings suggest that hypertension plays a critical role in the development of advanced glomerular lesions in diabetic eNOS −/− mice and that eNOS deficiency induces aldosterone breakthrough, reducing the efficacy of RAS blockades for DN. On the other hand, Yuen et al. have shown that ACEI (captopril) remarkably suppresses albuminuria in low-dose STZ eNOS −/− mice, concomitant with reduction of BP [39]. They also demonstrated that albuminuria in this model is prominently reduced by ARB (losartan) at a dose that does not lower BP and that spironolactone is less effective than captopril or losartan. Thus, low-dose STZ eNOS −/− mice showed distinct responses to RAS blockade. Further, we have shown that antihypertensive triple therapy (hydralazine + hydrochlorothiazide + reserpine) or captopril normalizes BP and remarkably reduces glomerular and tubulointerstitial injury in db/db eNOS −/− mice [41]. Interestingly, captopril exhibited more profound effects in db/db eNOS −/− mice than antihypertensive therapy, whereas reduction of BP was comparable in both therapies, suggesting additive eNOS-independent renoprotective effects of ACEI in DN. Collectively, these studies indicate that hypertension has a strong impact on renal injury in diabetic eNOS −/− mice and that the RAS system may serve as a key mediator for this, yet ACEI or ARB failed to reduce BP and renal injury in high-dose STZ eNOS −/− mice (this might be related to STZ toxicity).

**Figure 2:**
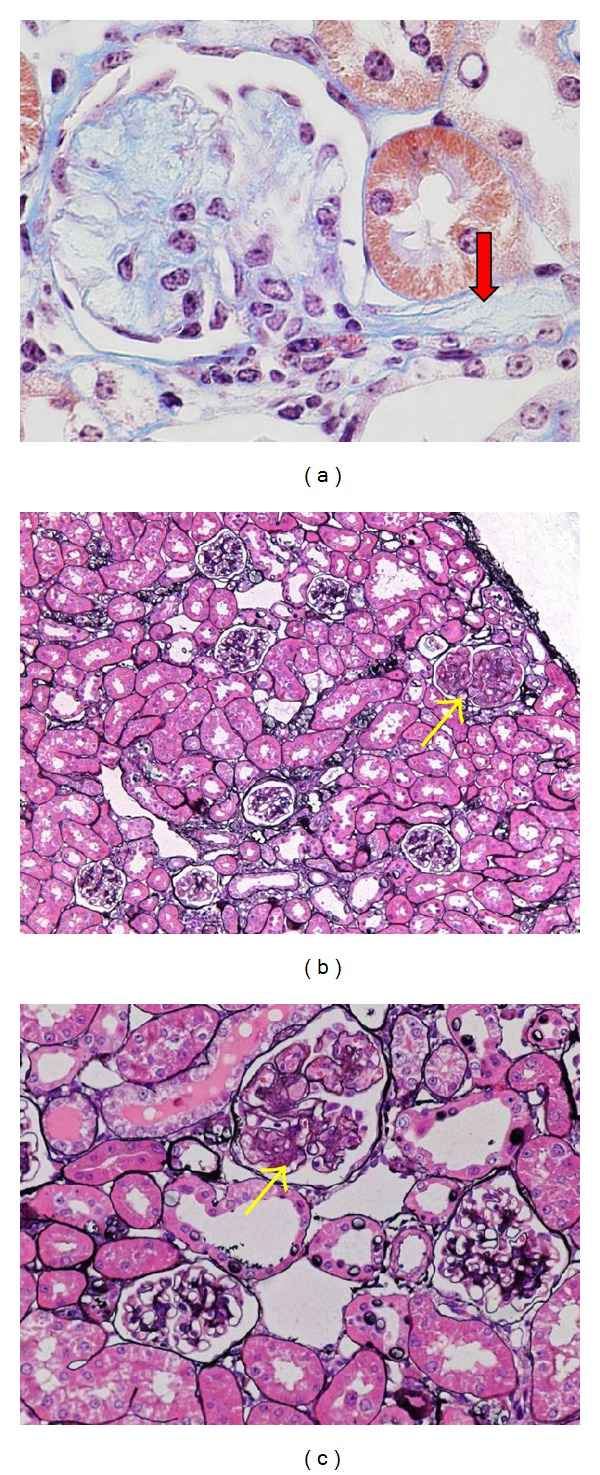
Glomerular mesangiolysis in STZ eNOS −/− mice at 22 weeks after low-dose STZ injections. (a) Mesangiolysis is accompanied by afferent arteriole injury (arrow). (b and c) Prominent mesangiolysis (arrows) is observed in subpopulation of glomeruli, indicating heterogeneity of glomerular injury in this model. Note adjacent glomeruli exhibit mild glomerular lesion.

Diabetic eNOS −/− mice are hypertensive; however, the elevation of BP is mild~moderate in these mice, especially in STZ models (Table 1). Why does antihypertensive or RAS blockade treatment has such a strong impact on glomerular injury in diabetic eNOS −/− mice? The eNOS-derived NO was shown to alter intraglomerular pressure and perfusion by modulating autoregulation or by counteracting Ang II vasoconstriction in afferent and efferent arteries [68, 69]. Therefore, an underlying mechanism could be (1) barotrauma to the glomerulus due to disruption of autoregulation and/or dominant vasoconstriction of efferent arteries and (2) glomerular hypoperfusion due to vasoconstriction of afferent or renal arteries [69]. In this context, it is of note that Nakagawa et al. have reported that inner lumen size in afferent arteries is increased in STZ eNOS −/− mice, particularly in the glomerulus with mesangiolysis [33]. In addition, we noted that mesangiolysis frequently accompanies afferent arteriolar lesions (Figure 2(a)). These findings suggest that eNOS deficiency may impair autoregulation concomitant with afferent arteriolar injury in diabetic mice, leading to hyperperfusion of glomeruli. In addition, it should be noted that the severity of glomerular injury largely differs among individual glomeruli in diabetic eNOS −/− mice (Figures 2(b) and 2(c)); 5–10% of glomeruli exhibited prominent mesangiolysis, while others do not. Although the mechanism of this large heterogeneity of glomerular injury is currently unknown, this finding may indicate that the degree or mode of arteriolar injury differs in individual glomeruli, possibly associated with the location in the renal circulation or sensitivity of the glomerulus to Ang II. Indeed, prominent mesangiolysis was more frequently observed in outer region of renal cortex (~80% in outer half of cortex and ~20% in inner half of cortex) in STZ eNOS −/− mice (Kanetsuna Y. unpublished observation). Thus, further investigation would be required to determine the effects of eNOS deficiency in glomerular (and renal) hemodynamics and autoregulation in diabetes.

Renal as well as systemic renin-angiotensin system (RAS) is known to play a key role in DN. How does eNOS deficiency alter renal RAS in diabetes? Interestingly, intrarenal Ang II (protein) levels were decreased or unaltered in eNOS −/− mice with STZ diabetes, while diabetes increased renal Ang II in eNOS +/+ mice [39, 46]. Thus, the levels of renal Ang II were lower in STZ eNOS −/− mice than in STZ eNOS +/+ mice. This finding suggests that eNOS is required for the increase in renal Ang II in diabetes and that increased Ang II sensitivity or responses of the target cells may be a responsible mechanism of RAS activation in this model. Further, alteration of renal RAS components was examined in more detail in high-dose STZ eNOS −/− and db/db eNOS −/− models [41, 46]. In high-dose STZ model, renal expression of RAS components was changed in a similar manner in STZ eNOS −/− and eNOS +/+ mice compared with nondiabetic counterparts as follows: (upregulated) angiotensinogen mRNA, ACE2, AT1 receptor, and MCR proteins, (downregulated) renin mRNA, and (unaltered) ACE protein. (Note that, in this study, the levels of RAS components were not compared between STZ eNOS −/− and eNOS +/+ mice.) In db/db eNOS −/− and db/db kidneys, RAS components were changed in the following manner compared with nondiabetic mice: (upregulated) angiotensinogen mRNA, (downregulated) renin and ACE1 mRNA, and (unaltered) ACE2 and Ang II receptors (AT1a, AT1b, AT2, and Mas). The db/db eNOS −/− kidney showed significantly higher angiotensinogen and lower renin mRNA expression compared with db/db eNOS +/+ kidney. Similar results were obtained with the glomeruli of these mice, yet pronounced downregulation of renin mRNA was not observed in db/db eNOS −/− glomeruli and ACE1 mRNA was unaltered in db/db eNOS −/− and eNOS +/+ glomeruli. Also, Mas mRNA was comparably downregulated in db/db eNOS +/+ and eNOS −/− glomeruli. Thus, diabetes alters renal RAS components in a similar manner in diabetic eNOS −/− and eNOS +/+ mice and the pronounced changes in renal angiotensinogen and renin mRNA were noted in db/db eNOS −/− mice. However, it is still unclear how eNOS deficiency alters renal RAS activity during the course of DN. Further investigation would be required on this subject. Endothelial NO is also known to reduce endothelin-1 production and its action, a key pathogenic pathway in DN. However, the role of this pathway in this model still remains unknown.

*Enhanced VEGF Signaling and Renal Angiogenesis*. Abnormal angiogenesis has been implicated in the pathogenesis of DN, coupled with increased production of angiogenic factors such as VEGF and angiopoietin-2 [70, 71]. Interestingly, Nakagawa et al. have demonstrated that inhibition of NO synthesis enhances VEGF-induced endothelial cell proliferation through VEGFR2-ERK1/2 pathway in normal and high glucose culture conditions [72]. Further, the increased endothelial proliferation (in glomerulus and peritubular capillaries) and enhanced VEGFR2 phosphorylation were noted in diabetic eNOS −/− kidney, compared with diabetic eNOS +/+ kidney, yet significant differences were not observed in mRNA levels of renal VEGF and VEGFR2 between these two groups [33, 37, 39]. Increased endothelial cell proliferation and VEGFR2 phosphorylation were also noted in nondiabetic eNOS −/− kidney [33, 39]; however, its extent was limited in these mice, suggesting that diabetes or diabetes-mediated VEGF upregulation is required for this effect. Given the fact that eNOS activity is increased by VEGF in most circumstances, Nakagawa et al. proposed that “VEGF-eNOS uncoupling” may be a mechanism that causes excessive VEGF signaling and angiogenesis in DN [73]. Further, more recently, Veron et al. demonstrated that overexpression of VEGF in podocytes causes nodular glomerulosclerosis, mesangiolysis, microaneurysms, and arteriolar hyalinosis concurrent with massive proteinuria and renal failure in eNOS −/− mice in the absence of diabetes [74], suggesting that increased VEGF activity and eNOS deficiency are sufficient to develop advanced DN-like lesions in mice. This is a very interesting model. However, some questions remain to be answered. First, the detailed mechanisms by which eNOS deficiency enhances VEGFR2 signaling and endothelial proliferation have not been determined yet. In addition, a body of studies has shown that eNOS deficiency “inhibits” VEGF-mediated angiogenesis in various conditions including cancer [67, 75, 76], indicating that eNOS is a critical downstream mediator of VEGF angiogenesis. Why does eNOS deficiency show a distinct effect in renal microvasculature, especially in the setting of diabetes? Is this effect specific for renal endothelium or diabetes? Second, it is also uncertain whether the enhanced VEGF signaling and angiogenesis is a responsible mechanism of advanced DN in diabetic eNOS −/− mice. Yuen et al. have recently shown that a VEGFR inhibitor (vatalanib) is ineffective in reducing albuminuria in STZ eNOS −/− mice, while it effectively decreases albuminuria in STZ eNOS +/+ mice [39]. This study also demonstrated that VEGFR inhibition is ineffective in reducing mesangial expansion and GBM thickening in STZ eNOS −/− mice, though it effectively suppressed glomerular capillary growth in this model. These findings suggest that (1) the enhanced VEGFR activity accounts for a part of, but not all, the glomerular phenotype in STZ eNOS −/− mice. (2) Unlike other diabetic mouse models, diabetic albuminuria is caused by a VEGF-independent mechanism in eNOS −/− mice. Podocytopathy was suggested as a mechanism of this [39]. (3) VEGF-mediated glomerular capillary growth occurs in diabetic mice in the absence of eNOS. In this context, it should be noted that eNOS deficiency was shown to alter morphogenesis of capillary growth and results in less sprouting and enlarged vessels [77]; therefore, the morphogenesis of glomerular capillary growth in diabetic eNOS −/− mice may differ from those in DN where eNOS activity is partially decreased. Thus, further investigation would be required to determine whether VEGF-eNOS deficiency induces the pathological signals that are analogous to progressive DN. (Note that NO inhibition or eNOS knockdown was shown to upregulate VEGF or VEGFR2 expression in endothelial cells [73, 78]. This may be another mechanism by which eNOS deficiency enhances VEGF signaling, although there was no difference in total VEGF and VEGFR2 mRNA levels between diabetic eNOS −/− and eNOS +/+ kidneys [33, 39].)

*Podocytopathy*. Yuen et al. have recently shown that diabetic eNOS −/− mice (STZ and db/db models) develop podocytopathy soon after the onset of diabetes, associated with the development of albuminuria, while podocyte injury is limited in diabetic eNOS +/+ or nondiabetic eNOS −/− mice [39]. The podocytopathy was observed in STZ eNOS −/− mice as early as 2 weeks after STZ injections and in db/db eNOS −/− mice as early as 8–12 weeks of age. Further, this study showed that (1) the podocytopathy and albuminuria in STZ eNOS −/− mice are largely suppressed by captopril or losartan at a dose that does not lower BP. (2) The soluble factors, which are produced in diabetic eNOS −/− mice or in cultured eNOS −/− glomerular endothelial cells exposed to high glucose and Ang II, remarkably alter the cell shape and cytoskeleton arrangement in cultured podocytes, concomitant with an increase in RhoA activity. These findings suggest that eNOS deficiency confers RAS-dependent acute podocytopathy to diabetic mice and that this podocytopathy may be caused by endocrine or paracrine mechanisms. Identification of the responsible factors should give new insights into the pathogenesis of DN. For this topic, it was also shown that Nicorandil reduces the podocyte oxidative stress and loss in STZ eNOS −/− mice through ATP-dependent K channel activation [43].

*Impaired Glomerular Endothelial Barrier and Integrity*. A body of studies has shown that endothelial surface glycocalyx, a negatively charged surface layer of membrane-associated proteoglycans and glycosaminoglycans, serves as an important charge barrier in glomerular filtration, and its disruption may cause albuminuria [79]. Using cationic ferritin, we have shown that the anionic surface barrier in glomerular endothelium is damaged in STZ eNOS −/− mice [32]. This mechanism is also supported by the following evidence: (1) chronic NO inhibition impairs glomerular endothelial charge barrier concomitant with albuminuria and GBM thickening [80]. (2) The disrupted glomerular charge barrier in eNOS −/− mice was demonstrated by a recent MRI study using cationic ferritin as a contrast agent [81]. Further, we noted that heparanase expression is upregulated in the glomerulus (endothelial and mesangial cells) of STZ or db/db eNOS −/− mice (Takahashi T, unpublished observation). Given the fact that heparanase impairs surface glycocalyx in glomerular endothelial cells [82], these findings suggest that NO deficiency may upregulate heparanase expression in glomerulus in the setting of diabetes, disrupting the endothelial surface glycocalyx, and lead to albuminuria. Since glomerular heparanase expression was shown to be associated with the development of DN in humans as well as in mice [83, 84], it would be of interest to examine the role of eNOS in the expression of heparanase and other glycocalyx disrupting enzymes in diabetic glomeruli.

Endothelial intercellular junctions also constitute an important barrier in vasculature. Disruption of this barrier causes the influx of serum proteins to the subendothelial space, inducing vascular cellular responses and facilitating the formation of advanced vascular lesions. We have noted that immunoreactivity of VE-cadherin is decreased in the glomerular endothelium of low-dose STZ eNOS −/− mice [32]. Although the precise mechanism of this is currently unknown, this finding suggests that eNOS deficiency may impair glomerular endothelial junctions in diabetes, causing insudation to subendothelial space and mesangium, and advance diabetic glomerular lesions.

*Inflammatory Gene Expression*. NO is known to inhibit inflammation. Consistent with this finding, enhanced inflammatory gene expression was observed in diabetic eNOS −/− or eNOS +/− kidneys, compared with diabetic eNOS +/+ kidney [34, 35, 41]. These include TNF*α*, IL-6, MCP-1, ICAM-1, VCAM-1, and iNOS. In addition, it was shown that eNOS deficiency and high-fat diet synergistically increases renal expression of IL-1*β* and IL-6 in STZ-induced diabetic mice [34]. Given the fact that eNOS deficiency itself had no effects on renal inflammatory gene expression [34], these findings suggest that eNOS deficiency accelerates renal inflammatory gene expression in the setting of diabetes.

*HB-EGF*. We have recently demonstrated that renal expression of heparin-binding epidermal growth factor-like growth factor (HB-EGF) is upregulated in both db/db eNOS −/− and nondiabetic eNOS −/− mice as early as at 8 weeks of age, including arterial and glomerular endothelium, podocytes, and tubular cells [85]. HB-EGF expression was higher in db/db eNOS −/− mice and its levels were further increased during the progression of nephropathy, accompanied by EGFR phosphorylation and urinary HB-EGF excretion. Further, we also demonstrated that (1) L-NAME treatment dramatically increases renal HB-EGF expression and its urinary excretion in db/db eNOS +/+ mice. (2) Replenishing NO with sodium nitrate reduces renal HB-EGF expression and its urinary excretion in db/db eNOS −/− mice and inhibits the progression of nephropathy. (3) Genetic deletion of endothelial HB-EGF expression attenuates glomerular injury in STZ eNOS −/− mice. These findings suggest that NO deficiency induces HB-EGF expression in diabetic kidney and this may serve as an important mediator of progressive DN. In addition, we have recently shown that the renal phenotype in STZ eNOS −/− mice is remarkably suppressed by an EGFR inhibitor (erlotinib) [53]. Of interest, this was accompanied by activation of AMPK pathway, inhibition of mTOR pathway, decreased renal ER stress, and increased autophagy. Taken together, these findings indicate a pivotal role of EGF pathway in this pathological condition. Further investigation of this pathway should provide a new insight into the pathogenesis of DN.

*Toll-Like Receptor TLR4*. Lin et al. have recently shown that STZ eNOS −/− kidney exhibits enhanced expression of toll-like receptor (TLR) TLR4 and its ligand HMGB1, predominantly in tubular cells, and that synthetic TLR4 antagonist, CRX-526, significantly attenuates renal injury in this model without altering blood glucose and systolic blood pressure, including albuminuria, renal hypertrophy, and glomerular and tubulointerstitial injury [40]. Further, the authors demonstrated that CRX-526 treatment decreases induction of chemokine (C-C motif) ligand (CCL)-2, osteopontin, CCL-5, TGF-*β*, and NF-*κ*B activation in STZ eNOS −/− kidney and reduces macrophage infiltration and collagen deposition in glomerulus and interstitium. These results suggest that eNOS deficiency accelerates renal TLR4 pathway in diabetes and promotes renal inflammation and fibrosis.

*Coagulation and Platelet Activation*. NO inhibits coagulation and platelet aggregation and activation. Consistent with this finding, diabetic eNOS −/− or eNOS +/− glomeruli showed increased thrombus formation that was accompanied by fibrin deposition and platelet accumulation [32–35, 37] and this was further enhanced by a high-fat diet [34]. Glomerular fibrin deposition was also observed in nondiabetic eNOS −/− mice; however, its extent was highly limited in these mice [33, 37], indicating that eNOS deficiency promotes glomerular thrombus formation in the setting of diabetes. Interestingly, Li et al. demonstrated that renal tissue factor (TF) mRNA levels and activity are increased in diabetic eNOS −/− and eNOS +/− mice compared with diabetic eNOS +/+ mice in Akita or STZ models, and these mice exhibited prominent TF immunoreactivity in the mesangial area [34, 35]. Further, TF and MOMA-2 coimmunostaining indicated that all TF-positive cells are monocyte/macrophages. The authors also demonstrated that TF levels correlate with albuminuria, GBM thickening, and tubulointerstitial fibrosis; TF expression is increased before the development of DN; TF neutralizing antibody prominently decreases renal expression of inflammatory and fibrogenic genes in diabetic mice regardless of eNOS genotype; high-fat diet additively increases renal TF levels and activities [34]. An increase in renal TF expression was also observed in nondiabetic eNOS −/− mice; however, it was relatively limited. In aggregate, these findings suggest that eNOS deficiency promotes TF expression in diabetic glomeruli through monocyte/macrophage infiltration and this activates the coagulation cascade in the mesangial area, promoting inflammatory and fibrogenic gene expression and accelerating glomerular injury. In addition, a recent study has demonstrated that eNOS −/− glomeruli show increased von Willebrand factor (vWF) deposition both in glomerular capillaries and the mesangial area [86]. Given the fact that serum P-selectin levels are increased in eNOS −/− mice, suggesting exocytosis of Weibel-Palade body by the endothelium, the authors proposed a model that eNOS deficiency causes endothelial release of vWF, resulting in mesangial as well as intraluminal deposition of vWF and facilitating glomerular thrombus formation and mesangial expansion. Although diabetic eNOS −/− mice were not investigated in this study, the finding suggests that a similar event may occur in diabetic eNOS −/− glomeruli.

*Oxidative Stress*. A large body of study has shown that increased oxidative stress plays a central role in the pathogenesis of DN. Hence the effects of eNOS deficiency on renal oxidative stress have been investigated in some studies. In the low-dose STZ model, oxidative stress (plasma and renal CML and urinary 8-OHdG) in diabetic eNOS −/− mice was comparable to those in diabetic eNOS +/+ mice [34], yet eNOS deficiency and high-fat diet synergistically increased oxidative stress (plasma CML and urinary 8-OHdG) in STZ diabetic mice. Similar results were also observed in our STZ study (Kanetsuna Y, unpublished observation); both STZ eNOS +/+ and eNOS −/− mice exhibited increased renal oxidative stress (nitrotyrosine and 8-OHdG staining) as compared with nondiabetic counterparts. However, significant differences were not observed between these two groups. Surprisingly, renal oxidative stress was significantly reduced in Akita eNOS −/− and eNOS +/− mice compared with Akita eNOS +/+ or nondiabetic eNOS −/− mice [35]. In contrast, db/db eNOS −/− kidney showed pronounced nitrotyrosine and 4-HNE staining [41]. Although a comparison between db/db eNOS +/+ and db/db eNOS −/− kidneys has not been performed, the finding suggests increased renal oxidative stress in this model. Thus, the effects of eNOS deficiency on diabetic renal oxidative stress seem to be different among the models and oxidative stress may not be a key mechanism in low-dose STZ and Akita eNOS −/− models.

*Other Potential Mechanisms*. (1) A recent study showed that blockade of the Ca2+-activated K+ channel KCa3.1, which is expressed in proximal tubules, reduces albuminuria, renal hypertrophy, and the expression of inflammatory and fibrotic markers in tubulointerstitium in STZ eNOS −/− mice without affecting blood pressure levels [45], suggesting involvement of KCa3.1 pathway in this model. (2) NO was shown to regulate the expression and activity of hypoxia-inducible factor (HIF1), a key mediator in kidney disease, through multiple mechanisms in physiological and pathological conditions [87, 88]. Also, eNOS deficiency may lead to vasoconstriction, reducing renal blood flow, and cause renal hypoxia. Therefore, HIF-1 may mediate renal phenotype in diabetic eNOS −/− mice. Although difference was not observed in renal HIF-1*α* mRNA levels between STZ eNOS −/− and eNOS +/+ mice [34], further investigations should be performed on this subject, including HIF1 protein level, S-nitrosylation, and its activity. (3) Lipotoxic disruption of Na+/H+ exchanger NHE1 interaction with PI(4,5)P2 was recently implicated in proximal tubule apoptosis in db/db eNOS −/− mice [89].

**Figure 3:**
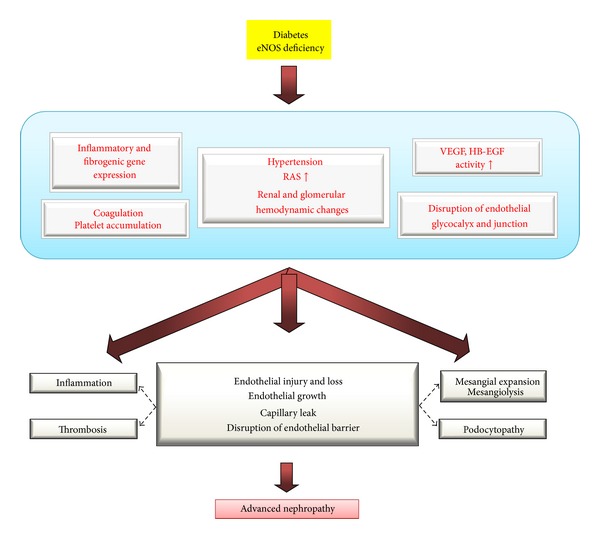
Pathogenesis of advanced DN in diabetic eNOS −/− mice. Abbreviations: RAS, renin-angiotensin system; VEGF, vascular endothelial growth factor; HB-EGF, heparin-binding epidermal growth factor-like growth factor.

In summary, multiple mechanisms are involved in the development of advanced DN in diabetic eNOS −/− mice (Figure 3). Among them, hypertension and RAS system seem to play a major role in this model as in human DN.

## 4. Future Directions

The study of this animal model will be highly valuable with the following aspects. First, this model, especially db/db eNOS −/− mice, develops glomerular lesions of progressive DN. Second, the nephropathy in this model progresses as the mice age, as indicated by declining GFR and increasing glomerulosclerosis. Third, the pathophysiology (decreased eNOS activity) underlying this model is relevant to human DN. In addition, the glomerular gene expression profile in db/db eNOS −/− mice was recently shown to overlap to those in human DN [90]. Thus, this model provides an invaluable tool for us to study the eNOS-mediated pathogenic mechanisms underlying progressive DN. On the other hand, we may also need to realize that there are some differences between this animal model and advanced human DN. First, VEGF and nephrin expression were shown to be downregulated in advanced human DN, concurrent with podocyte detachment and reduced glomerular capillary endothelial fenestration [91–93]. However, glomerular VEGF expression is upregulated in this model [44] and endothelial fenestration and nephrin expression are still well preserved [32, 39] (Advani A, unpublished observation). Although the changes of glomerular VEGF and nephrin expression have not been evaluated in db/db eNOS −/− model, given the fact that glomerular gene expression in db/db eNOS −/− mice overlaps to those in early human DN [90], this model seems to still display early DN or a transition phase to advanced DN. Second, it is well known that diabetes causes eNOS uncoupling and the uncoupled eNOS serves as a major source of superoxide overproduction in diabetic endothelium [15]. In addition, NO was shown to promote superoxide generation by reducing oxygen consumption in mitochondria [94, 95]. Because these superoxide generations do not occur in eNOS deficient mice, the endothelial oxidative stress in eNOS-deficient diabetic mice may be lower than that in actual diabetic endothelium. Further, eNOS deficiency may decrease the production of reactive nitrogen species (e.g., peroxynitrite) that is harmful to the cells. These might be a reason why renal oxidative stress and tubulointerstitial lesion are decreased in Akita eNOS −/− mice. Last, the mesangiolysis seen in diabetic eNOS −/− mice is more severe than is often seen in human DN. Its morphological features are similar to thrombotic microangiopathy rather than DN, indicating severe glomerular endothelial injury. Thus, there are some differences in pathophysiology of renal injury between diabetic eNOS −/− mice (eNOS deficiency-mediated) and actual DN (eNOS dysfunction). Therefore, it would be very important to validate the findings obtained from this model with human subjects. In this context, heterozygous model may serve better than homozygous model and the creation of a diabetic mouse model in which eNOS is uncoupled would be of great interest.

**Figure 4:**
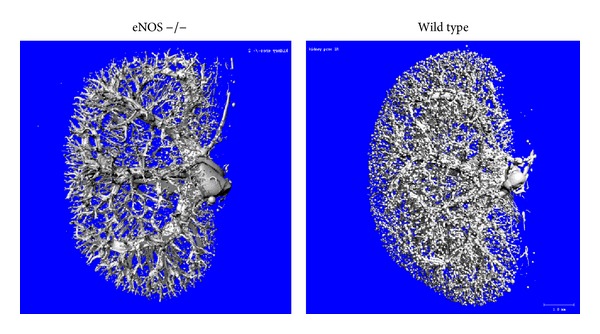
MicroCT assessment of renal vasculature in eNOS −/− mice. Three dimensional microCT renderings of contrast (Microfil) perfused renal vasculature in 8 week-old eNOS −/− and wild type mice are shown. Perfusable vessels are remarkably decreased in eNOS −/− mouse kidney. The data displays representative data of ten mice.

The following efforts and investigation would be required to better understand the role of eNOS in DN. First, the reported STZ eNOS −/− studies have been done on the nephropathy-resistant genetic background (C57BL/6J strain). In addition, the genetic background (C57BL/6J × 129 SvEv F1) of Akita eNOS −/− mice seems to also be nephropathy-resistant as the urinary albumin excretion in Akita eNOS +/+ mice was less than 100 *μ*g/day even at 7 months of age. Therefore, it would be required to conduct those studies with nephropathy-prone strain, such as DBA2/J, C57BL6 × DBA2 F1 hybrid, or 129SvEv [51, 96], to determine the precise effects of eNOS deficiency in DN that was caused by hypoinsulinemic diabetes. Since STZ is toxic to eNOS −/− mice, it would be desirable to conduct these studies with Akita model. High-dose STZ model should not be used for future works. Also, the findings obtained with low-dose STZ model should be confirmed with Akita model. Second, there are many unresolved issues with the reported models: (1) the cause of death in diabetic eNOS −/− mice is not defined, yet occasional bleeding in the chest and abdominal cavity and clots in the mesenteric arteries and necrosis of small intestine [34, 35] suggest that hypercoagulability may in part contribute to premature death of these mice. (2) Akita eNOS −/− study has emerged a role for eNOS to “negatively” regulate diabetic hyperfiltration, whereas previous studies proposed a role of eNOS as a positive regulator of diabetic hyperfiltration based on the effects of nonselective NOS inhibitors. However, the mechanism of this is currently unknown. Also, to date, it is unclear whether db/db eNOS −/− mice exhibit pronounced (or reduced) diabetic hyperfiltration at early age. (3) eNOS deficiency reduced oxidative stress and tubulointerstitial fibrosis in Akita mice. However, the underlying mechanisms remain unknown. (4) db/db eNOS −/− mice developed prominent hyperinsulinemia; however, the mechanism of this is currently unknown. Since eNOS deficiency was shown to reduce insulin sensitivity [97, 98], this may be due to the enhanced insulin resistance in these mice. Third, eNOS −/− mice have been shown to exhibit various congenital vascular anomalies with high frequency, including congenital septal defects and postnatal heart failure [99], abnormal aortic valve development [100], and defects in pulmonary vascular development [101]. Further, eNOS −/− mice were shown to exhibit congenital renal defects including renal scars containing crowded small glomeruli [102] and progressive renal disease [86]. In addition, we have recently noted that the number of perfusable renal vessels is remarkably decreased in eNOS −/− mice compared with wild type mice (Figure 4). Thus, the eNOS −/− kidney exhibits significant vascular and renal lesions without diabetes. Therefore, it is still unclear whether “eNOS deficiency accelerates DN” or “diabetes accelerates the renal injury in eNOS −/− mice.” Also, eNOS is expressed in various cell types including cardiomyocytes, hematopoietic cells such as erythrocytes and platelets, adipocytes, and renal tubular epithelial cells [103, 104]. Consequently, the precise role of eNOS in diabetic endothelium or vasculature is not defined clearly. The postnatal conditional knockout study to target endothelial or vascular (endothelial and hematopoietic) eNOS in diabetic mice would be required to better understand the role of eNOS in DN. For this reason, we have recently generated a floxed eNOS mouse [105]. This mouse would be useful for addressing this issue. Last, a body of studies has shown a critical role of eNOS-derived NO in regulation of renal hemodynamics and oxygen consumption. Further, the fact that antihypertensive or RAS inhibitor treatment remarkably reduces glomerular injury in diabetic eNOS −/− mice suggests that hemodynamic events may play a key role in the pathology of this model. However, it is still unclear how eNOS deficiency alters renal or glomerular hemodynamics and oxygenation in diabetic mice and what factors are involved in these changes. In addition, cardiac phenotype is not investigated in detail, yet significant changes were not observed in the pulse levels between db/db eNOS −/− and eNOS +/+ mice [37].

## 5. Conclusions

In recent decade, several groups have generated diabetic eNOS −/− models, either with hypoinsulinemic or type 2 diabetes, and defined the critical role of eNOS in the development of advanced diabetic renal lesions. Further, the efforts to investigate the underlying mechanisms in this model explored unreported eNOS-associated pathogenic mechanisms in this disease. The complete eNOS deficiency does not occur in actual DN; therefore, diabetic eNOS −/− model may include some artificial events that are irrelevant to DN. However, given the facts that this animal model was developed through a mechanism that is relevant to human DN and exhibits the most advanced diabetic renal lesion among the reported mouse models, further investigations of this model should largely advance our understanding of the pathogenesis of progressive DN and these studies enable a more efficient interrogation of novel therapeutics for this disease.

## Figures and Tables

**Table 1:** General phenotype of diabetic eNOS −/− mice.

Phenotype	Models			
	High-dose STZ	Low-dose STZ	Akita	db/db
Hyperglycemia∗	→	→	→	↓
Survival rate (at 5-6 months after diabetes)	50%	70–100%	50%	?
Body weight∗	↓	→	↑	↑
Hypertension	+	+	+	+
(SBP increases, mm Hg)∗	23	10–24	26 (at 7 mo)	35–48
Hyperlipidemia∗	?	(−)	?	+ (Cho↑)
Reference #	[33]	[32, 34, 40]	[35]	[36, 37]

*Vs. diabetic wild-type mice; mo, months; Cho, plasma cholesterol; SBP, systolic blood pressure.

**Table 2:** Comparison of renal phenotype in diabetic eNOS −/− mice.

Phenotype	Models			
	High-dose STZ (C57BL/6J)	Low-dose STZ (C57BL/6J)	Akita (C57BL/6J × 129S6/SvEv F1)	db/db (C57BLKS/J)
Albuminuria				
Vs. DM WT mice	19.2 (fold increase)	1.8–8.7	2.5	5.3–5.7
Vs. non-DM WT mice	43.1	2.0–30	16	34.9–37.5
GFR∗	**↓↓ **	↓	**↑↑ **	**↓↓**
Tubulointerstitial lesion∗	++	+	decreased	++
Oxidative stress∗	?	→	↓↓	↑↑
Sensitivity to RAS blockade	(−)	(+)	?	(+)
Reference #	[33]	[32, 34, 39, 40]	[35]	[36, 37, 41]

*Vs. diabetic wild-type (DM WT) mice.
